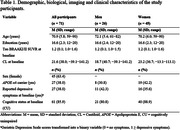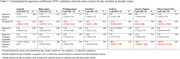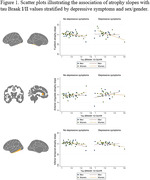# Medial temporal lobe vulnerability in women: the interactive effect of tau and depressive symptoms on atrophy trajectories

**DOI:** 10.1002/alz.091480

**Published:** 2025-01-09

**Authors:** Eleni Palpatzis, Pablo Aguillar, Muge Akinci, Eider M Arenaza‐Urquijo

**Affiliations:** ^1^ Barcelona Institute for Global Health (ISGlobal), Barcelona, Barcelona Spain; ^2^ Universitat Pompeu Fabra, Barcelona Spain; ^3^ ISGlobal, Barcelona, ‐ Spain; ^4^ Universitat Pompeu Fabra, Barcelona, Barcelona Spain; ^5^ 3Sant Pau Memory Unit, Hospital de la Santa Creu i Sant Pau, Biomedical Research Institute Sant Pau, Barcelona, Barcelona Spain; ^6^ ISGlobal ‐ Barcelona Institute for Global Health, Barcelona, Catalunya/Barcelona Spain

## Abstract

**Background:**

Women show greater atrophy in medial temporal lobe (MTL) compared to men, irrespective of amyloid burden. We examined whether depressive symptoms, which are more prevalent in women and also linked to MTL atrophy, interact with tau accumulation on MTL atrophy trajectories across 7 years differently among men and women.

**Method:**

We included 71 non‐demented ADNI participants with available baseline data on depressive symptoms (Geriatric Depression Scale; GDS), MTL tau accumulation (AV1451 PET BRAAK I/II SUVR [entorhinal and hippocampus]), Centiloids derived from [^18^F]florbetaben and [^18^F]florbetapir and with at least 3 structural MRI visits (mean time = 6.8 years, SD = 2.1). Individual slopes indicating GM volume change of MTL structures (hippocampus, parahippocampus, entorhinal cortex, fusiform and amygdala) were calculated with linear‐mixed effects models. To assess the specificity of the results to the MTL, the inferior temporal lobe and the anterior cingulate (ACC) were selected as control regions. Presence of depressive symptoms was measured with GDS (0 = no symptoms, 1≥depressive symptoms). We performed linear regression models to examine the effects of tau accumulation, depressive symptoms and the sex/gender‐related effects of tau*depressive symptoms on MTL, inferior temporal and ACC atrophy trajectories. Covariates included baseline age, years of education, total intracranial volume and baseline amyloid burden.

**Result:**

63% of participants were women and there were no differences in presence/absence of depressive symptoms by sex/gender (Table 1). Tau accumulation was associated with longitudinal atrophy in all brain regions while presence of depressive symptoms was not (Table 2). The presence of depressive symptoms interacted with tau in a sex/gender‐specific manner on fusiform, ACC and inferior temporal lobe, such that, women who reported depressive symptoms had higher atrophy rates as a function of tau accumulation compared to men (Figure 1).

**Conclusion:**

Our findings indicate that the presence of depressive symptoms amplify the impact of tau on GM atrophy trajectories in women. Contrary to expectations, the results do not substantiate a specific vulnerability in the MTL, but rather reveal a broader susceptibility encompassing tau‐related inferior temporal areas and the anterior limbic system. In women, depressive symptoms may contribute to regional brain vulnerability downstream amyloid deposition by amplifying the effects of tau.